# Association of calprotectin with other inflammatory parameters in the prediction of mortality for ischemic stroke

**DOI:** 10.1186/s12974-020-02047-1

**Published:** 2021-01-05

**Authors:** Juan Marta-Enguita, Manuel Navarro-Oviedo, Idoia Rubio-Baines, Nuria Aymerich, Maria Herrera, Beatriz Zandio, Sergio Mayor, Jose-Antonio Rodriguez, Jose-Antonio Páramo, Estefania Toledo, Maite Mendioroz, Roberto Muñoz, Josune Orbe

**Affiliations:** 1 Neurology Department, Complejo-Hospitalario de Navarra, Pamplona, Spain; 2 Atherothrombosis Laboratory, Program of Cardiovascular Diseases, CIMA-Universidad de Navarra, IdiSNA, Pio-XII, 55, 31008 Pamplona, Spain; 3 CIBERCV, ISCIII, Madrid, Spain; 4 Preventive Medicine and Public Health Department, Universidad de Navarra, IdiSNA, Pamplona, Spain; 5 CIBEROBN, ISCIII, Madrid, Spain; 6 Neuroepigenetics Laboratory-Navarrabiomed, Complejo-Hospitalario de Navarra, Universidad Pública de Navarra-UPNA, IdiSNA, Pamplona, Spain

**Keywords:** Calprotectin, Mortality, Stroke, S100a9 protein, Biomarker

## Abstract

**Background:**

Inflammatory response plays an important role in many processes related to acute ischemic stroke (AIS). Calprotectin (S100A8/S100A9), released by monocytes and neutrophils, is a key protein in the regulation of inflammation and thrombosis. The purpose of this study is to evaluate the association of circulating calprotectin with other inflammatory biomarkers and AIS prognosis, as well as the calprotectin content in stroke thrombi.

**Methods:**

Among the 748 patients treated at a comprehensive stroke center between 2015 and 2017, 413 patients with confirmed acute ischemic injury were prospectively evaluated. Patients with systemic inflammation or infection at onset were excluded. Plasma calprotectin was measured by ELISA in blood samples of AIS patients within the first 24 h. Univariate and multivariate logistic regression models were performed to evaluate its association with mortality and functional independence (FI) at 3 months (defined as modified Rankin Scale < 3) and hemorrhagic transformation (HT) after ischemic stroke. Further, S100A9 was localized by immunostaining in stroke thrombi (*n* = 44).

**Results:**

Higher calprotectin levels were associated with 3-month mortality, HT, and lower 3-month FI. After adjusting for potential confounders, plasma calprotectin remained associated with 3-month mortality [OR (95% CI) 2.31 (1.13–4.73)]. Patients with calprotectin ≥ 2.26 μg/mL were 4 times more likely to die [OR 4.34 (1.95–9.67)]. Addition of calprotectin to clinical variables led to significant improvement in the discrimination capacity of the model [0.91 (0.87–0.95) vs 0.89 (0.85–0.93); *p* < 0.05]. A multimarker approach demonstrated that patients with increased calprotectin, CRP, and NLR had the poorest outcome with a mortality rate of 42.3% during follow-up. S100A9 protein, as part of the heterodimer calprotectin, was present in all thrombi retrieved from AIS patients. Mean S100A9 content was 3.5% and tended to be higher in patients who died (*p* = 0.09). Moreover, it positively correlated with platelets (Pearson *r* 0.46, *p* < 0.002), leukocytes (0.45, *p* < 0.01), and neutrophil elastase (0.70, *p* < 0.001) thrombus content.

**Conclusions:**

Plasma calprotectin is an independent predictor of 3-month mortality and provides complementary prognostic information to identify patients with poor outcome after AIS. The presence of S100A9 in stroke thrombi suggests a possible inflammatory mechanism in clot formation, and further studies are needed to determine its influence in resistance to reperfusion.

## Introduction

Stroke is the most frequent cause of permanent disability in adults and one of the most important causes of death [1]. Over the next years, global stroke burden is expected to increase steadily, mainly because of population aging [2]. Ischemic stroke remains to represent more than 80% of all strokes. Even after years of research, pathophysiology of brain ischemia and post-ischemic changes in the brain are not yet fully understood. Inflammation is increasingly recognized as a key element in the pathological progression of ischemic stroke. Brain ischemia causes an immediate local immuno-inflammatory reaction with strong activation of microglia, astrocytes, and endothelial cells and release of cytokines both from activated cells and endothelium [3]. This non-specific response after brain tissue damage results in blood-brain barrier permeability and infiltration of inflammatory cells into the ischemic area [4] which have the potential to further increase tissue injury. Likely, multiple inflammatory molecules have been reported as predictive markers of stroke severity and outcome and proposed as potential therapeutic targets to be modulated as a neuroprotective strategy in acute ischemic stroke (AIS) [5]. 

Neutrophil-to-lymphocyte ratio (NLR), easily calculated by white blood cell count on admission, has been reported as a prognostic biomarker in stroke. High admission NLR values predict 3-month clinical outcome in thrombectomized patients [6] and are associated with symptomatic hemorrhagic transformation (HT) after AIS [7]. C-reactive protein (CRP), a sensitive indicator of inflammation rapidly produced by the liver after tissue injury or infection [8], is one of the most widely used markers in clinical practice. In AIS patients, CRP levels have been reported to be significantly higher than controls in all ischemic stroke subtypes [9].

Calprotectin is a heterodimer formed by two cytosolic proteins, S100A8 and S100A9, expressed by white blood cells, especially monocytes and neutrophils. Also known as myeloid-related protein-8 and -14 (MRP8/14), calprotectin plays an important role in promoting inflammation and is a validated marker of disease activity in inflammatory bowel disease and rheumatoid arthritis [10]. Studies focusing on coronary events have shown that plasma calprotectin was related to first and future coronary events, independently of traditional cardiovascular risk factors and C-reactive protein [11]. Recently, circulating calprotectin was associated with increased peripheral artery disease risk prediction, further improved when combined with high sensitivity CRP [12]. Moreover, plasma calprotectin has been proposed as a diagnostic marker of AIS [13], and inhibition of S100A9 has been reported to suppress thrombus formation in experimental models of stroke [14, 15].

Nevertheless, calprotectin prognostic role and its association with other inflammatory biomarkers, such as CRP and NLR, have not yet been studied in stroke patients. The purpose of this study is to evaluate circulating calprotectin levels as a prognosis biomarker in AIS and to analyze how it relates to other well-known inflammatory biomarkers. Furthermore, we have studied the composition of ischemic stroke thrombi and characterized S100A9 content, to assess its association with thrombus components and clinical parameters.

## Methods

### Study population

**Fig. 1:**
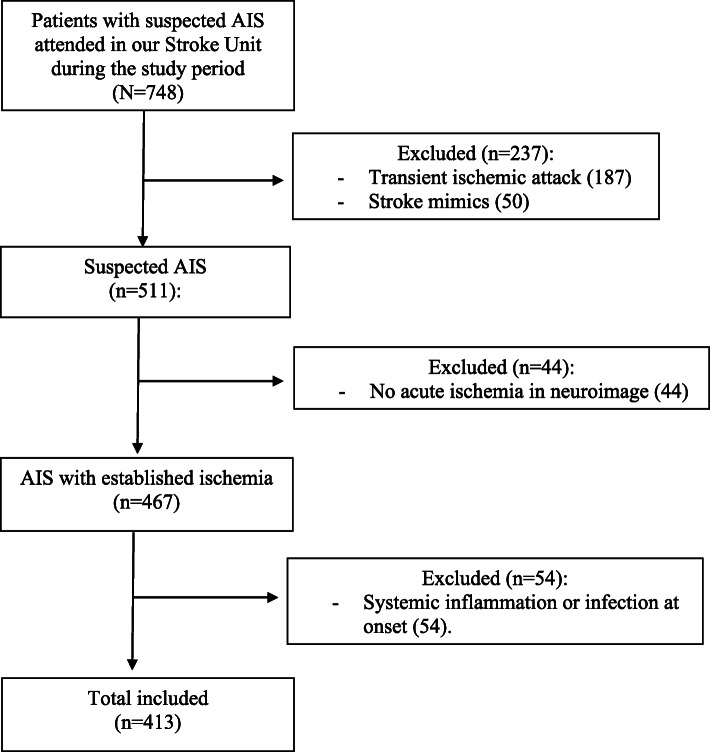
Flowchart of the study

This is a single-center prospective study of consecutive AIS patients admitted to our Stroke Unit of the Complejo Hospitalario de Navarra between November 2015 and November 2017. All patients with acute neurologic deficits (< 24 h) were included when AIS was suspected and written informed consent was obtained (*n* = 748). Patients with transient ischemic attack and stroke mimics were excluded and only those with established acute ischemic injury confirmed by neuroimaging (mainly diffusion-weighted MRI and some of them delayed cranial CT) were included in this study. Patients with systemic inflammation or infection at onset (including active cancer, post-operative patients, and active systemic infections) were excluded to avoid confusion with previous inflammatory condition (Fig. 1).

### Clinical information

Baseline characteristics of the study patients, including demographics (age, sex), weight, estimated glomerular filtration rate (eGFR), previous cardiovascular disease, vascular risk factors, serum glucose, systolic and diastolic blood pressure (SBP and DBP, respectively) at admission, previous use of antithrombotic agents (antiplatelet agent and anticoagulants), stroke severity assessed by National Institutes of Health Stroke Scale (NIHSS) and treatment with tissue-type plasminogen activator (tPA) and/or mechanical thrombectomy, were recorded. The vascular risk factors included were hypertension (patients taking antihypertensive drugs or with blood pressure > 140/90 mmHg on repeated measurements), type 2 diabetes (patients taking antidiabetic drugs, fasting blood sugar ≥ 126 mg/dL or HbA1c ≥ 6.5%, or a casual plasma glucose > 200 mg/dL), hypercholesterolemia [patients receiving lipid-lowering agents or with an overnight fasting cholesterol level ≥ 240 mg/dL, triglycerides ≥ 200 mg/dL, or low-density lipoprotein (LDL) cholesterol ≥ 160 mg/dL], and current cigarette smoking. Assessment of kidney function was based on estimated eGFR calculated using the Chronic Kidney Disease Epidemiology Collaboration creatinine equation. Etiologic subtypes of ischemic stroke were classified based on the Trial of Org 10172 in Acute Stroke Treatment (TOAST) classification [16] that was reviewed at 3 months after an exhaustive search of paroxysmal atrial fibrillation which often includes a prolonged cardiac monitoring.

A CT scan was obtained at admission for all patients, and the Alberta Stroke Program Early CT Score (ASPECTS) was collected by two independent radiologists. CT scan was repeated at 24–48 h in those patients who underwent mechanical thrombectomy and/or received tPA in order to evaluate infarct area and identify HT. MRI scans were obtained within 1 week of stroke onset using a 1.5-T MR imaging unit if not contraindicated (delayed CT scan was elective when MRI was contraindicated). HT after AIS was identified on this follow-up neuroimaging and characterized by a stroke specialized neurologist.

### Sample processing and plasma calprotectin measurement

Venous blood samples were drawn from all patients in the emergency department before the initiation of any treatment as standard-of-care, and neutrophils, lymphocytes (Alinity hq, Abbott, USA), and serum glucose (Architect i2000SR, Abbott, USA) were measured by standard laboratory techniques. After receiving or not treatment, new blood samples were taken within the following 24 h and C-reactive protein (CRP) was measured with an autoanalyzer (Architect i2000SR, Abbott, USA). These second samples were centrifuged at 1200×*g* for 15 min, 4 °C within 2 h of collection, and stored at -80°C for further analysis. Citrated plasma samples were thawed on ice and thoroughly vortexed before measuring calprotectin levels (LEGEND MAX Human MRP8/14 ELISA Kit, BioLegend, USA) with an automated ELISA analyzer TRITURUS (Grifols, Spain). The inter- and intra-assay variability averages were 7.4% and 2.8%, respectively. All experiments were performed in a blinded manner following the manufacturer’s instructions.

### Histological analysis

Retrieved thrombi from thrombectomized IS patients (*n* = 44) were transferred to saline until fixed in formalin (PanReac AppliChem, Spain) during 24 h. Then, samples were embedded in paraffin by a tissue automatic processor (Tissue-Tek VIP, Sakura, Japan) and paraffin-embedded clot material was cut into 3-μm sections with a rotatory microtome (HM-340E, Microm, Germany). Serial slides from each thrombus were stained with hematoxylin and eosin (PanReac AppliChem, Spain), with Martius Scarlet Blue staining (Atom, UK) and with specific antibodies against platelets (CD42b, Invitrogen, USA), neutrophil elastase (NE, Sigma-Aldrich, USA), and the calcium-binding protein S100A9 (PA5-82145, Invitrogen, USA) as part of the heterodimer calprotectin. Immunostained slides were scanned (Aperio ImageScope, Leica ByoSistems, Germany) and quantified with ImageJ software [17]. Data are presented as the percentage of positive stained area in the total tissue area.

### Outcome measures

Main clinical outcomes were defined by modified Rankin Scale (mRS) score at 90 days, established by face-to-face interview with a stroke specialized neurologist. The primary clinical outcome was 3-month all-cause mortality. Secondary outcomes included 3-month functional independence, defined as 90-day mRS < 3, and HT after ischemic stroke including hemorrhagic infarcts (HI type 1 or 2), parenchymal hematomas (PH type 1 or 2), and remote hematomas or subarachnoid hemorrhages, according to the European Cooperative Acute Stroke Study III (ECASS III) classification [18]. Patients without follow-up regarding stroke-related functional outcome after 90 days were excluded from the analysis for FI (*n* = 21).

### Statistical analysis

Continuous variables with normal distributions were presented as mean with standard deviation (SD), while non-normally distributed variables were presented as median with interquartile range (IQR). Normality of distributions was assessed graphically and with the Shapiro-Wilk test. Logarithmic transformation was applied for continuous variables with skewed distributions. Continuous variables were compared between groups using the unpaired *t* test or the Wilcoxon rank-sum test depending on their distribution. Comparisons between binary categorical variables were performed using the chi-square test or, in the case of small-expected frequencies, Fisher’s exact test. Pairwise Spearman correlations were performed between continuous variables to evaluate correlation. Stroke subtype classification was assessed by TOAST criteria, and dichotomized etiological groups were created. NIHSS score was categorized ([0–7], [7–14], and [> 14]), and analysis of variance and trend analysis were performed.

Association of baseline characteristics with outcomes was performed first with univariate logistic regression models. To evaluate 3-month functional independence (mRS < 3), patients with an initial qualifying status (mRS score > 2) were excluded (*n* = 50). Multivariate logistic regression models were performed considering as potential confounders those traits which yielded a *p* value < 0.10 in the univariate analyses or those traits whose inclusion led to clinically relevant changes (OR variations > ± 10%). In addition, variables that have been identified as important covariates for poor outcome after ischemic stroke in previous studies were also included. Selected multivariate binary logistic regression models were performed to evaluate associations between calprotectin with clinical outcomes after adjustment for potential confounders. Results were expressed as odds ratios (ORs) with 95% confidence intervals (95% CI). Interactions were evaluated with the likelihood-ratio test, and stratified models were generated if the rendered *p* values were below 0.05. The Homer-Lemeshow test was used to assess the calibration of the models. *K*-fold cross-validation was performed to test the model’s ability to predict new data that was not used in estimating it [19].

Complementarily, to evaluate predictive models for 3-month mortality and 3-month FI in our population, baseline characteristics associated with a value of *p* < 0.20 in univariate analyses were implemented in a forward-stepwise multivariate logistic regression model with a significance level for addition to the model of *p* < 0.05.

Receiver operating characteristic (ROC) curve analysis, with binary logistic regression, was used to determine the discrimination capacity of calprotectin, NIHSS, CRP, and NLR as outcome predictors. ROC analysis was used to compare the complete model for each outcome with and without calprotectin. Then, we calculated the integrated discrimination improvement (IDI) to evaluate the improvement in the model’s calibration after adding calprotectin to the 3-month mortality multivariate model, both as a continuous and a dichotomous (cut-off point 2.26 μg/mL) trait [20, 21]. Besides, a cut-off point for each of the inflammatory markers tested (NLR, CRP, and calprotectin) was rendered by ROC analysis, and mortality predictions were performed according to the presence of none, 1, 2, or 3 increased markers as defined by these cut-offs.

For all analyses, *p* < 0.05 was considered statistically significant. All analyses were performed with the STATA software (version 14.2, StataCorp LLC, TX, USA).

## Results

A total of 413 patients with AIS confirmed by neuroimaging were finally included for the analysis. The mean age of our population was 75.6 years (SD 12.4 years), 71.4% were hypertensive and 27.1% diabetic. Among this elderly population, around 11.9% of patients had baseline mRS score ≥ 3. Stroke severity was mild with median NIHSS score of 6 points (IQR 3–15). HT incidence was 12.4% and mortality rate was 17.1%. Baseline characteristics of the study patients are presented in Table 1.

**Table 1:** Baseline characteristics of the study population

Variable	*n* = 413
Age, years^a^	75.6 (12.4)
Female^c^	176 (42.6)
Weight, kg^b^	72.5 (64.5–82)
Hypertension^c^	295 (71.4)
Type 2 diabetes^c^	112 (27.1)
Dyslipidemia^c^	201 (48.7)
eGFR < 45 mL/min/1.73 m^2 c^	39 (9.5)
Antiplatelet therapy^c^	129 (31.2)
Anticoagulant therapy^c^	86 (20.8)
SBP at admission, mmHg^b^	152 (135–171)
DBP at admission, mmHg^b^	82 (73–93)
Serum glucose at admission, mg/dL^b^	113 (99.5–142)
Neutrophil count at admission, × 10^9^/L^b^	5.2 (4.1–6.7)
Lymphocyte count at admission, × 10^9^/L^b^	1.8 (1.3–2.4)
Baseline NIHSS score^b^	6 (3–15)
ASPECTS < 7^c^	16 (4.01)
ASPECTS, points^a^	9.45 (1.24)
Baseline mRS score^c^	
mRS 0	164 (39.8)
mRS 1	125 (30.3)
mRS 2	74 (18.0)
mRS 3	32 (7.8)
mRS 4	17 (4.1)
Etiologic subtype by TOAST^c^	
LAA	43 (10.4)
SVO	62 (15.0)
Cardioembolic stroke	178 (43.1)
Undetermined	123 (29.8)
Other causes	7 (1.7)
Posterior circulation stroke^c^	55 (13.3)
Intravenous thrombolysis^c^	151 (36.6)
Endovascular treatment^c^	70 (17.0)
Hemorrhagic transformation^c^	51 (12.4)
3-month mortality^c^	67 (17.1)
3-month functional independence^c^	227 (57.9)

*SD* standard deviation, *IQR* interquartile range (denoted by 25th–75th percentile), *eGFR* estimated glomerular filtration rate, *SBP* systolic blood pressure, *DBP* diastolic blood pressure, *NIHSS* National Institute of Health Stroke Scale, *ASPECTS* Alberta Stroke Program Early CT Score, *mRS* modified Rankin Scale, *LAA* large-artery atherosclerosis, *SVO* small-vessel occlusion

^a^Continuous variables with normal distributions are presented as means (SD)

^b^Continuous non-normally distributed variables are presented as medians (IQR)

^c^Categorical variables are presented as *n* (%)

The most common etiology by TOAST was cardioembolic (*n* = 178, 43%), followed by small-vessel occlusion (*n* = 62, 15%), large-artery atherosclerosis (*n* = 43, 10.4%), and other causes (*n* = 7, 1.7%) with 30% of strokes with undetermined etiology (*n* = 123).

### Plasma calprotectin levels by outcomes

**Fig. 2:**
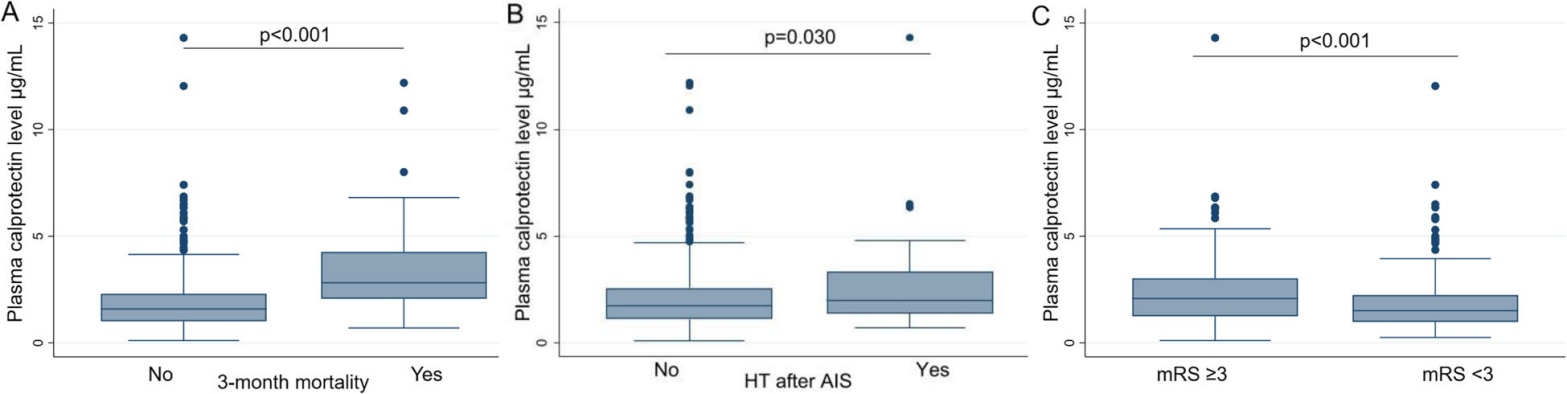
Box-plots of plasma calprotectin levels by outcomes. Differences in **a** 3-month mortality, **b** HT after AIS, and **c** 3-month FI. HT, hemorrhagic transformation; AIS, acute ischemic stroke; mRS; modified Rankin Scale; FI; functional independence. Calculated by Wilcoxon rank-sum test

Median plasma calprotectin level was 1.76 μg/mL (IQR, 1.14–2.66). As shown in Fig. 2a, patients who died within 3 months showed higher calprotectin levels [median (IQR) μg/mL, 2.81 (2.06–4.26) vs 1.58 (1.00–2.29), *p* < 0.001], as did patients who developed HT after AIS [1.99 (1.36–3.32) vs 1.72 (1.10–2.56), *p* = 0.030, Fig. 2b]. Furthermore, lower plasma calprotectin levels after AIS were observed in patients with 3-month FI [1.49 (0.97–2.23) vs 2.2 (1.41–3.38), *p* < 0.001, Fig. 2c].

### Relationship between calprotectin and clinical and analytical variables

Plasma calprotectin levels correlated with neutrophil (Pearson *r* 0.28, *p* < 0.001) and lymphocyte count (− 0.17, *p* = 0.007), NLR (0.29, *p* < 0.001), CRP (0.50, *p* < 0.001), and fibrinogen (0.27, *p* = 0.001).

Calprotectin levels were significantly associated with clinical stroke severity by NIHSS score in the ANOVA test (*p* < 0.001), and a significant linear trend was observed (*p* < 0.001) (Additional file 1: Supplementary Figure 1A).

When analyzing clinical factors, a positive correlation with age was registered (Pearson *r* 0.18, *p* < 0.001) with a significant linear trend (*p* = 0.012) in the ANOVA test. In addition, the mRS score at baseline was also associated with plasma calprotectin levels (*p* < 0.02) with a significant positive linear trend (*p* < 0.001). Besides, higher calprotectin levels [1.97 (1.51–3.94) vs 1.71 (1.10–2.59), *p* < 0.02] were observed in patients with eGFR < 45 mL/min/1.73 m^2^.

Patients with confirmed cardioembolic stroke—according to TOAST criteria—showed higher calprotectin levels than patients with non-cardioembolic stroke [1.97 (1.23–2.97) vs 1.66 (0.99–2.39), *p* < 0.004] (Additional file 1: Supplementary Figure 1B).

### Predictors of mortality at 90 days

Patients who died within 90 days were older (median age, 86.6 vs 75.7 years; *p* < 0.001), more frequently females (58.2 vs 41.8%; *p* = 0.05), had higher DBP (median DBP, 87.5 vs 82.9 mmHg; *p* = 0.04), had worse NIHSS score (median NIHSS score, 17 vs 4; *p* < 0.001), mRS at admission (median mRS, 1.94 vs 0.91; *p* < 0.001), and a lower ASPECTS (median ASPECT score, 8.5 vs 9.6; *p* < 0.001) than patients who survived at 90 days.

Univariate analysis confirms sex, age, hypertension and DBP, eGFR < 45 mL/min/1.73 m^2^, NIHSS score, baseline mRS, cardioembolic stroke, and ASPECT score association with mortality (Table 2). In addition, intravenous treatment with tPA was associated with a higher 3-month mortality and a higher incidence of HT after stroke. Elevated inflammatory markers (neutrophil count, NLR, CRP, and calprotectin) were associated with a higher risk of mortality. After adjusting for potential confounders, plasma calprotectin levels remained associated with 3-month mortality [per a log+1 increase OR (95% CI) 2.31 (1.13–4.73)] (Table 2). However, NLR and CRP did not remain associated with 3-month mortality in multivariate analysis [per a log+1 increase: OR 1.43 (0.77–2.63) and 1.09 (0.74–1.61), respectively]. No significant interactions were observed, and no stratified models were generated. This predictive model had a good calibration as indicated by the Hosmer-Lemeshow goodness-of-fit test (*p* value = 0.93). The averaged measure of fitness in *k*-fold cross-validation with *k* = 5 was 0.31 for this model (range 0.22–0.39).

**Table 2:** Univariate and multivariate logistic regression models of 3-month mortality with calprotectin and other baseline characteristics

Variable	Univariate logistic regression		Multivariate logistic regression (*n* = 333)	
	OR (95% CI)	*p* value	OR (95% CI)	*p* value
Age (per 10-year increase)	2.21 (1.65–2.97)	< 0.001	1.71 (1.05–2.79)	0.033
Sex (female)	2.09 (1.23–3.56)	0.007	1.04 (0.41–2.63)	0.939
Weight (kg)^a^	0.25 (0.06–1.03)	0.055	0.60 (0.05–7.03)	0.683
Hypertension	2.95 (1.41–6.18)	0.004	2.70 (0.85–8.53)	0.091
Type 2 diabetes	0.64 (0.34–1.21)	0.168		
Dyslipidemia	0.66 (0.39–1.13)	0.131		
eGFR < 45 mL/min/1.73 m^2^	2.56 (1.22–5.37)	0.013	1.40 (0.48–4.10)	0.542
SBP at admission^a^ (per 10-mmHg increase)	2.35 (0.48–11.48)	0.290		
DBP at admission (per 10-mmHg increase)	1.17 (1.00–1.36)	0.048	^b^	
Serum Glucose (mg/dL)^a^	1.97 (0.88–4.44)	0.101	1.79 (0.45–7.18)	0.410
Neutrophil count (_x_mm3)^a^	3.64 (1.83–7.24)	< 0.001	^b^	
Lymphocyte count (_x_mm3)^a^	0.42 (0.24–0.72)	0.002	^b^	
NLR^a^	2.41 (1.63–3.58)	< 0.001	1.43 (0.77–2.63)	0.254
C-reactive protein^a^ (mg/L)	1.02 (1.01–1.03)	< 0.001	1.09 (0.74–1.61)	0.645
Calprotectin μg/mL^a^	4.60 (2.86–7.40)	< 0.001	2.31 (1.13–4.73)	0.022
NIHSS score				
8–14 (vs 0–7)	3.75 (1.60–8.80)	0.002	3.01 (1.02–8.87)	0.046
> 14 (vs 0–7)	11.86 (5.85–24.05)	< 0.001	6.67 (2.25–19.82)	0.001
ASPECTS ≥ 7	0.08 (0.03-0.24)	< 0.001	0.09 (0.01–0.64)	0.017
Baseline mRS score (vs mRS0)			1.67 (1.17–2.37)	0.004
mRS 1	1.82 (0.80–4.12)	0.152		
mRS 2	3.07 (1.32–7.17)	0.009		
mRS 3	20.15 (7.81–52.01)	< 0.001		
Intravenous thrombolysis	2.33 (1.37–3.98)	0.002	1.53 (0.61–3.82)	0.364
Endovascular treatment	1.49 (0.77–2.89)	0.240		
Hemorrhagic transformation	4.04 (2.10–7.77)	< 0.001	1.26 (0.40–3.94)	0.695
Cardioembolic stroke	2.37 (1.38–4.06)	0.002	0.71 (0.31–1.63)	0.413

*eGFR* glomerular filtration rate, *SBP* systolic blood pressure, *DBP* diastolic blood pressure, *NLR* neutrophil-to-lymphocyte ratio, *NIHSS* National Institute of Health Stroke Scale, *ASPECTS* Alberta Stroke Program Early CT Score, *mRS* modified Rankin Scale

^a^Log transformed

^b^Excluded to avoid collinearity between neutrophils, lymphocytes, and NLR and between HTA and DBP

As shown in Supplementary Table 1 (Additional file 2), after forward-stepwise selection of the strongest predictors, only age [per 10-year increase: OR (95% CI) 1.73 (1.18–2.53)], calprotectin [per a log+1 increase: OR 3.00 (1.71–5.26)], NIHSS score > 14 [OR 7.04 (3.08–16.10)], ASPECTS > 7 [OR 0.19 (0.05–0.77)], and baseline mRS score [OR 1.57 (1.14–2.15)] were independent predictors of 3-month mortality, whereas NLR and CRP were excluded from the analysis (*p* > 0.05). This predictive model had a good calibration as indicated by the Hosmer-Lemeshow goodness-of-fit test (*p* value = 0.51). The averaged measure of fitness in *k-*fold cross-validation with *k* = 5 was 0.30 for this model (range 0.23–0.37).

**Fig. 3:**
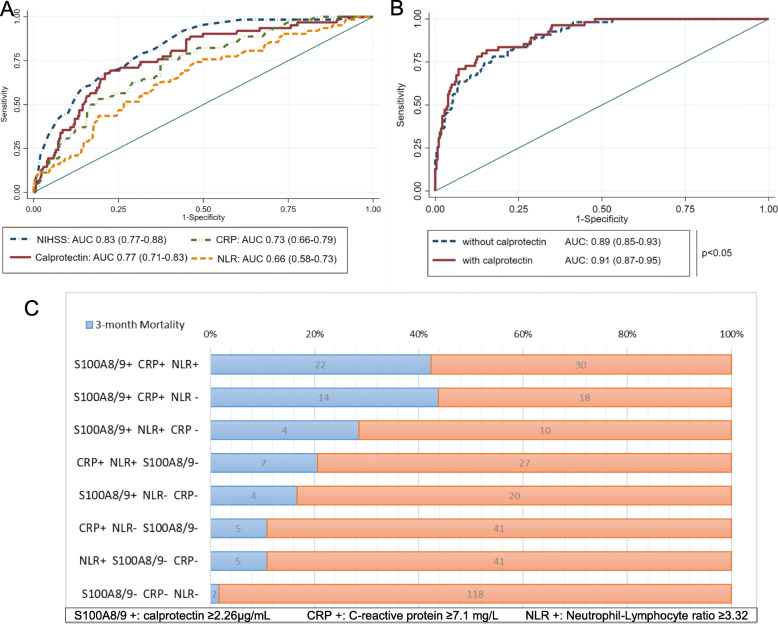
Prognostic value of stroke severity and inflammatory biomarkers to predict 3-month mortality. **a** ROC analysis of univariate regression models for 3-month mortality with NIHSS (blue), calprotectin (red), CRP (green), and NLR (orange). **b** ROC curves of a multivariate model of 3-month mortality with (red) and without calprotectin (blue), adjusted by age, sex, weight, eGFR, serum glucose, hypertension, stroke severity by NIHSS, iv tPA treatment, ASPECTS score > 7, cardioembolic stroke, baseline mRS, and hemorrhagic transformation after stroke. **c** 3-month mortality rates in our population stratifying according to the presence of 1, 2, or 3 inflammatory markers above the cut-off values. NIHSS, National Institute of Health Stroke Scale; CRP, C-reactive protein; NLR, neutrophil-to-lymphocyte ratio

ROC curve analysis for significant univariate models showed that the highest AUC was for NIHSS score [area under the curve (AUC) (95% CI), 0.83 (0.77–0.88)] followed by calprotectin as the strongest predictor of mortality across the inflammatory markers tested [AUC (95% CI) calprotectin 0.77 (0.71–0.83)] (Fig. 3a). The ROC analysis of the multivariate model rendered an AUC for 3-month mortality of 0.89 (95% CI 0.85–0.93). When calprotectin was added to the model, both the AUC [0.91 (0.87–0.95); *p* < 0.05 (Fig. 3b)] and the discrimination of poor outcome [IDI 0.045 (*p* < 0.002)] were improved. On the other hand, the addition to the multivariate model of NLR or CRP did not lead to a significant improvement in AUC [AUC for NLR 0.90 (0.86–0.94) and AUC for CRP 0.90 (0.87–0.94), both with *p* > 0.05]. Nevertheless, the differences in AUC were not statistically significant when we compared a multivariate-adjusted AUC including calprotectin with a multivariate-adjusted AUC including NLR (*p* = 0.32) or CRP (*p* = 0.41).

ROC analysis for calprotectin rendered a cut-off value of 2.26 μg/mL (sensitivity 72.7%; specificity 74.3%), which showed a negative predictive value of 92.9% in our population. When stratifying by this optimal cut-off and controlling for age, sex, weight, eGFR, hypertension, serum glucose, NIHSS score, baseline mRS, intravenous tPA administration, cardioembolic stroke, and ASPECTS, patients with calprotectin ≥ 2.26 μg/mL were 4 times more likely to die within 90 days than AIS patients with a calprotectin < 2.26 μg/mL [OR (95% CI) 4.34 (1.95–9.67)]. This addition of the dichotomized calprotectin by cut-off also improves discrimination of the best-fit model (IDI 0.053, *p* < 0.001).

Even though calprotectin levels seemed to better estimate mortality at 90 days, we assessed whether the combination with CRP and NLR might improve mortality prediction. Univariate ROC curves for CRP and NLR rendered cut-off values of 7.1 mg/L and 3.32, respectively. When considering the combination of these three parameters, patients with calprotectin ≥ 2.26 μg/mL, CRP ≥ 7.1 mg/L, and NLR levels ≥ 3.32 had an estimated probability of dying in the next 90 days of 49%, whereas calculated mortality risk in patients with negative readings for all 3 markers was 3.7%. Further, we stratified our study population according to the presence of none, 1, 2, or 3 of these inflammatory markers above the cut-off values (Fig. 3c). At 90 days, 1.6% of patients with negative readings had died. Also, 12.5% of patients with 1 positive marker, 30.7% of patients with 2 positive markers, and 42.3% of patients with 3 positive markers died.

### Predictors of 3-month functional independence

Variables associated with functional outcome in the univariate analysis were age, eGFR < 45 mL/min/1.73 m^2^, admission NIHSS score, cardioembolic stroke, baseline functional status by mRS score, intravenous tPA treatment, endovascular treatment, ASPECTS, and HT, as well as serum glucose, NLR, CRP, and calprotectin values (Table 3).

**Table 3:** Univariate and multivariate logistic regression models of 3-month functional independence with calprotectin and other baseline characteristics

Variable	Univariate logistic regression		Multivariate logistic regression (*n* = 302)	
	OR (95% CI)	*p* value	OR (95% CI)	*p* value
Age (per 10-year increase)	0.57 (0.46–0.71)	< 0.001	0.64 (0.47–0.88)	0.006
Sex (female)	0.67 (0.43–1.06)	0.089	0.80 (0.43–1.52)	0.502
Weight (kg)^a^	0.87 (0.26–2.93)	0.827		
Hypertension	0.74 (0.45–1.21)	0.232		
Type 2 diabetes	0.87 (0.53–1.42)	0.572		
Dyslipidemia	0.96 (0.61–1.50)	0.852		
eGFR < 45 mL/min/1.73 m^2^	0.44 (0.20–0.97)	0.041	0.70 (0.24–2.01)	0.508
SBP at admission^a^ (per 10-mmHg increase)	1.40 (0.36–5.36)	0.628		
DBP at admission (per 10-mmHg increase)	1.01 (0.89–1.16)	0.830		
Serum glucose at admission (mg/dL)^a^	0.34 (0.16–0.71)	0.004	0.28 (0.10–0.74)	0.011
Neutrophil count (_x_mm3)^a^	0.37 (0.20–0.66)	0.001	^b^	
Lymphocyte count (_x_mm3)^a^	2.40 (1.50–3.86)	< 0.001	^b^	
NLR^a^	0.44 (0.31–0.62)	< 0.001	0.62 (0.38–1.01)	0.055
C-reactive protein (mg/L)^a^	0.64 (0.53–0.78)	< 0.001	0.90 (0.68–1.18)	0.445
Calprotectin μg/mL^a^	0.49 (0.34–0.70)	< 0.001	1.04 (0.62–1.75)	0.877
NIHSS score at admission				
8–14 (vs 0–7)	0.16 (0.08–0.30)	< 0.001	0.12 (0.05–0.28)	< 0.001
> 14 (vs 0–7)	0.12 (0.07–0.21)	< 0.001	0.11 (0.04–0.31)	< 0.001
ASPECTS at baseline ≥ 7	18.95 (2.37–151.57)	0.006	5.03 (0.46–54.6)	0.185
Baseline mRS score ≤ 1	2.39 (1.40–4.06)	0.001	1.63 (0.80–3.30)	0.179
Intravenous thrombolysis	0.47 (0.30–0.74)	0.001	1.37 (0.64–2.93)	0.418
Endovascular treatment	0.51 (0.29–0.89)	0.018	1.84 (0.67–5.04)	0.237
Hemorrhagic transformation	0.13 (0.06–0.28)	< 0.001	0.23 (0.08–0.66)	0.007
Cardioembolic stroke	0.48 (0.32–0.73)	< 0.001	1.60 (0.83–3.08)	0.161

*eGFR* estimated glomerular filtration rate, *SBP* systolic blood pressure, *DBP* diastolic blood pressure, *NLR* neutrophil-to-lymphocyte ratio, *NIHSS* National Institute of Health Stroke Scale, *ASPECTS* Alberta Stroke Program Early CT Score, *mRS* modified Rankin Scale

^a^Log transformed

^b^Excluded by authors to avoid collinearity between neutrophils, lymphocytes, and NLR

In the multivariate analysis, none inflammatory marker was associated with 3-month FI after adjusting for potential confounders (Table 3). Nevertheless, when the analysis was repeated evaluating the strongest predictors by stepwise selection, only NLR remained associated with 3-month FI after adjusting for potential confounders [per a log+1 increase: OR (95% CI) 0.50 (0.33–0.77); *p* = 0.002] (Supplementary Table 2-Additional file 2). Univariate ROC analysis for 3-month FI showed a NLR cut-off point for FI of 2.71 [AUC (95% CI) 0.65 (0.58–0.71)]. When stratifying for this optimal cut-off and controlling for age, serum glucose, NIHSS score, and HT, patients with NLR ≥ 2.71 were 2.12 times more likely to be functional dependent at 90 days than AIS patients with an NLR < 2.71 [OR (95% CI) 2.12 (1.19–3.79)].

### Predictors of hemorrhagic transformation after ischemic stroke

Development of an HT after AIS was associated with admission NIHSS score, cardioembolic stroke, intravenous tPA treatment, and endovascular treatment, along with calprotectin and CRP in univariate analyses. Furthermore, HT was more frequent in females. Detailed results are presented in Additional file 2: Supplementary Table 3. However, in multivariate analyses only sex, NIHSS score ≥ 14, and intravenous tPA treatment remains statistically significant, and none inflammatory markers was independently associated with HT after AIS.

### Presence of S100A9 in thrombi retrieved from stroke patients

**Fig. 4:**
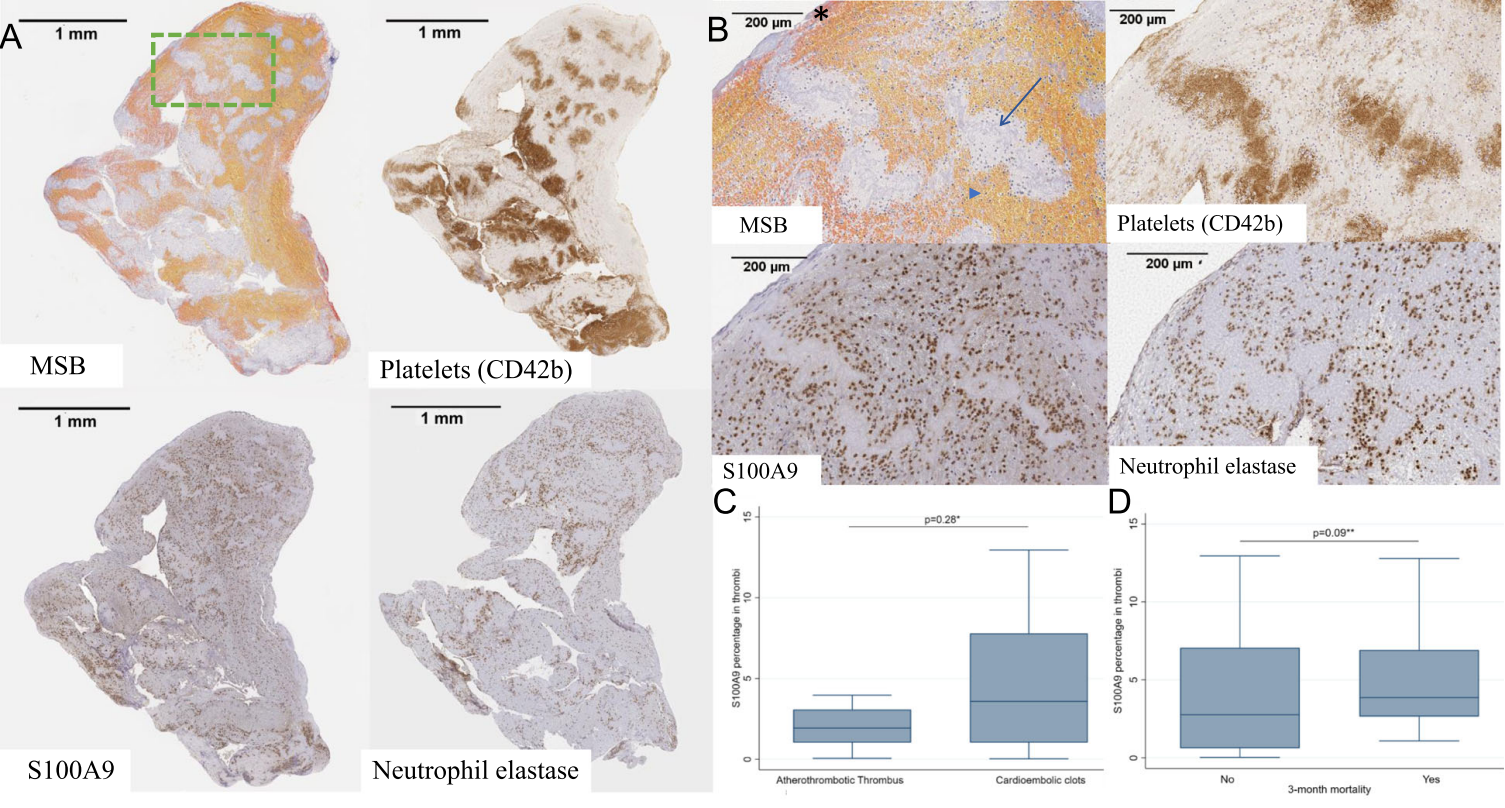
Histological staining and quantification of stroke thrombi. **a** Representative adjacent thrombus slides stained with Martius Scarlet Blue (MSB, left superior) and immunostained for platelets (CD42b), S100A9, and neutrophil elastase (in brown). **b** Magnifications of the area selected in panel **a**. MSB staining shows a red blood-rich area (orange-yellow, arrowhead) near to a collagen area (light blue, arrow). A fibrin area at the periphery appears in red (asterisk). Platelet-rich regions (CD42b staining in brown) are related with collagen areas. Neutrophil elastase (brown) tends to accumulate at the boundary of platelet-rich zones (brown with CD42b staining) and S100A9 distribution seems to be related with neutrophil and monocyte (neutrophil elastase +) presence at the interface between red blood cell-rich and platelet-rich areas and also within platelet islets. **c** Box-plot showing the differences in percentage of S100A9 staining in thrombi retrieved from cardioembolic and atherothrombotic stroke patients. Calculated by Wilcoxon rank-sum test. **d** Box-plot showing the differences of S100A9 percentage in thrombi from patients who died or survived after stroke. Calculated by Student *t* test over log-transformed S100A9

S100A9 protein was present in all thrombi analyzed and quantification of thrombus constituents revealed that stroke thrombi contained on average 12.8% (IQR 1.34–28.89) red blood cells, 15.05% (4.84–26.65) platelets, 0.62% (0.35–1.17) leukocytes, and 3.52% (1.17–6.68) S100A9. The distribution pattern of S100A9 through the thrombus seemed to be related with leukocyte distribution and was primarily found at the interface between red blood cell-rich and platelet-rich areas. Besides the described distribution, S100A9 was also present within platelet islets (Fig. 4a, b).

Interestingly, a positive correlation was observed between S100A9 and platelets (Pearson *r* 0.46, *p* < 0.002), leukocytes (0.45, *p* < 0.01), and neutrophil elastase (0.70, *p* < 0.001) thrombus content. When evaluating the amount of S100A9 in thrombi and its association with calprotectin circulating levels, we did not see a correlation. Furthermore, no correlation between thrombus S100A9 content and age, sex, functional outcome, and stroke severity was observed (not shown).

Finally, we observed a tendency to higher thrombi S100A9 amount in cardioembolic thrombi and in those who had died (Fig. 4c, d). The small sample size of atherothrombotic thrombi and the low number of deaths did not allow us to reach statistical significance (Additional file 2: Supplementary Table 4).

## Discussion

Our study shows that plasma calprotectin is a strong independent inflammatory predictor of 3-month mortality in AIS, and that improves the risk prediction of all-cause death associated to well-established factors such as stroke severity, high blood glucose, or baseline mRS. Out of inflammatory markers evaluated, plasma calprotectin showed the highest discrimination capacity for 3-month mortality. Furthermore, S100A9 as part of calprotectin heterodimer was present in all thrombus obtained by mechanical thrombectomy in stroke patients and seems to be higher in thrombi from patients who died and in those of cardioembolic etiology. However, calprotectin, CRP, and NLR were not independently associated with 3-month FI, or HT after AIS.

Calprotectin is produced by neutrophils and infiltrating macrophages in the ischemic brain [22]. Recently, in an experimental model of transient focal ischemia, inhibition of S100A9 reduced infarct volume and brain swelling [22] and a therapeutic vaccine against MRP14 (S100A9) resulted in thrombosis inhibition in a murine model of ischemic stroke [11]. In addition, calprotectin overexpression has been localized in ischemic hemisphere CD11b-positive cells [14], and brain proteomic analysis has shown that calprotectin was upregulated in experimental models of brain ischemia [22]. In plasma, it is a relatively stable and easily measurable protein and has been proposed as a potential biomarker of inflammatory processes. Calprotectin was previously shown as a useful biomarker of cardiovascular disease risk [23] and cardiovascular event and recurrence [24]. A very recent study of 4785 patients with AIS from 2 independent cohorts has reported that high plasma calprotectin concentrations at baseline were independently associated with poor prognosis and death within 3 months after ischemic stroke [25]. Our study confirms these results and extends previous data showing that higher calprotectin levels are associated with 3-month mortality in AIS patients with greater predictive power for mortality than other studied inflammatory markers.

CRP and NLR have been shown as potential biomarkers or therapeutic targets for stroke management [3, 26]. CRP is a sensitive indicator of inflammation [8] widely used in clinical practice. In a case-control study of 600 AIS patients, CRP levels were significantly higher for all ischemic subtypes than controls, both in the acute phase and at 3-month follow-up examinations [9]. Moreover, it has been reported that CRP was independent predictors of short-term outcome and mortality after AIS [27]. Likewise, higher NLR values have been shown as independent predictors of HT and 3-month mortality [6, 7] in patients with large vessel occlusion who underwent a mechanical thrombectomy. Besides, an early increase of neutrophils in patients with AIS has been associated with larger infarct volumes [28]. In our study, by contrast, CRP and NLR did not remain associated with 3-month mortality in adjusted models, particularly when calprotectin was included. However, NLR seems to be associated with FI in AIS patients acting as the strongest predictor for FI among the inflammatory markers tested. Our results showing a better prediction power of calprotectin for mortality, and its association with other inflammatory cells and proteins in our cohort, suggest that calprotectin might provide a useful complementary biomarker of immune-neutrophilic activation after AIS associated with worse outcomes.

In line with previous reports suggesting a multimarker approach to improve outcome prediction in AIS patients [29], our data indicate that calprotectin either alone or even better when combined with CRP and NLR could improve the overall prediction of 3-month mortality in AIS, suggesting that an elevated inflammatory state may contribute to the mortality in AIS patients. Though the predictive capacity of this multimarker approach in our cohort and the robust results on *K*-fold internal cross-validation, it will need further external validation in other cohorts of AIS patients.

Because little was known about the composition of human stroke thrombi, we assessed for the first time the presence of S100A9 in thrombi retrieved from stroke patients. S100A9 forms a heterodimer with S100A8 (Calprotectin) and has been reported as a key molecule in the regulation of thrombus formation [15]. It is expressed and secreted in blood by platelets and neutrophils after vascular injury [30]. Moreover, S100A9 gene knockout or neutralization reduces neutrophil recruitment and thrombotic effects by modulating platelet function without influencing other hemostatic parameters [12]. S100A9 is expressed in carotid [30], femoral atherosclerotic plaques [12], and in human coronary artery thrombus [14]. Our histological analysis showed that S100A9 is present in all ischemic stroke thrombi and correlated with inflammatory cells and platelets. According to our results, thrombus with confirmed cardiac source and from patients who died during 3-month follow-up seems to have a higher amount of S100A9 whereas no statistically significant differences were achieved due to a small sample size. Further studies are needed to elucidate whether S100A9 content or organization in stroke thrombi is associated with thrombus formation, resistance to revascularization therapies, and prognosis in stroke.

Our study provides further evidence about the potential role of plasma calprotectin on the prognosis of AIS. Besides, its presence in thrombus supports previous studies suggesting that plasma calprotectin could be implicated in arterial thrombosis process [31]. Evaluating calprotectin as a therapeutic target in ischemic stroke, other groups have shown that blocking calprotectin could reduce infarct volume, vascular inflammation, and improve neurologic deficits [14, 32, 33]. Therefore, further studies are needed to investigate whether calprotectin reduction with specific antagonists in the acute phase of AIS would improve AIS treatment strategies.

There are some limitations to this report. First, the modest sample size and the retrospective analysis of prospectively collected data are important methodological shortcomings. Second, the possibility of an unknown confounding factor cannot be ruled out completely. Third, calprotectin was measured in blood samples obtained at different times from onset for each patient (although always within 24 h after stroke onset). Fourth, our cohort presented a lower incidence of atherothrombotic etiology and a higher incidence of cardioembolic disease compared to previous studies. This fact might influence our results but could be partially explained due to an exhaustive search of cardioembolic source and an elderly population. Moreover, we have excluded from the study patients with systemic inflammation or infection at onset and, therefore, no major anti-inflammatory drugs are expected to be taken. Nevertheless, we cannot rule out that patients may have occasionally taken NSAIDs or other anti-inflammatory drugs before stroke onset. Finally, the observational study design did not allow establishing a cause-effect relationship.

## Conclusions

This study expands the existing evidence by showing that plasma calprotectin is independently related to 3-month mortality and could be used in clinical practice as a novel prognostic biomarker, providing complementary information to other inflammatory biomarkers in AIS patients. Further studies are needed to determine its influence in thrombus formation and resistance to reperfusion.

## Supplementary Information

**Additional file 1: Supplementary Figure 1**. **A**, Association of NIHSS score with plasma calprotectin levels expressed by median calprotectin levels stratified by NIHSS score at admission in three categories. **B**, Differences in calprotectin levels between cardioembolic and non-cardioembolic strokes. NIHSS: National Institute of Health Stroke Scale.

**Additional file 2: Supplementary Table 1**. Univariate and forward-stepwise multivariate logistic regression model of 3-month mortality. **Supplementary Table 2**. Univariate and forward-stepwise multivariate logistic regression model of 3-month functional independence. **Supplementary Table 3**. Univariate and multivariate logistic regression models of hemorrhagic transformation after AIS with calprotectin and other baseline characteristics. **Supplementary Table 4**. Characteristics of stroke patients with thrombi retrieved by mechanical thrombectomy.

## Data Availability

The data that support the findings of this study are available from the corresponding author on reasonable request.

## Abbreviations

AIS: Acute ischemic stroke
HT: Hemorrhagic transformation
FI: Functional independence
mRS: Modified Rankin Scale
NLR: Neutrophil-to-lymphocyte ratio
CRP: C-reactive protein
MRP8/14: Myeloid-related protein-8/-14
SBP: Systolic blood pressure
DBP: Diastolic blood pressure
NIHSS: National Institute of Health Stroke Scale
tPA: Tissue plasminogen activator
LDL: Low-density lipoprotein
TOAST: Trial of Org 10172 in Acute Stroke Treatment
ASPECTS: Alberta Stroke Program Early CT Score
LAA: Large-artery atherosclerosis
SVO: Small-vessel occlusion
MSB: Martius Scarlet Blue staining
SD: Standard deviation
IQR: Interquartile range
OR: Odds ratio
ROC: Receiver operating characteristics
