# Supplementary figures and images for: Association of calprotectin with other inflammatory parameters in the prediction of mortality for ischemic stroke
Source: J Neuroinflammation. 2021 Jan 5;18:3. doi: 10.1186/s12974-020-02047-1 (PMC7786493; doi:10.1186/s12974-020-02047-1)

## Slide 1
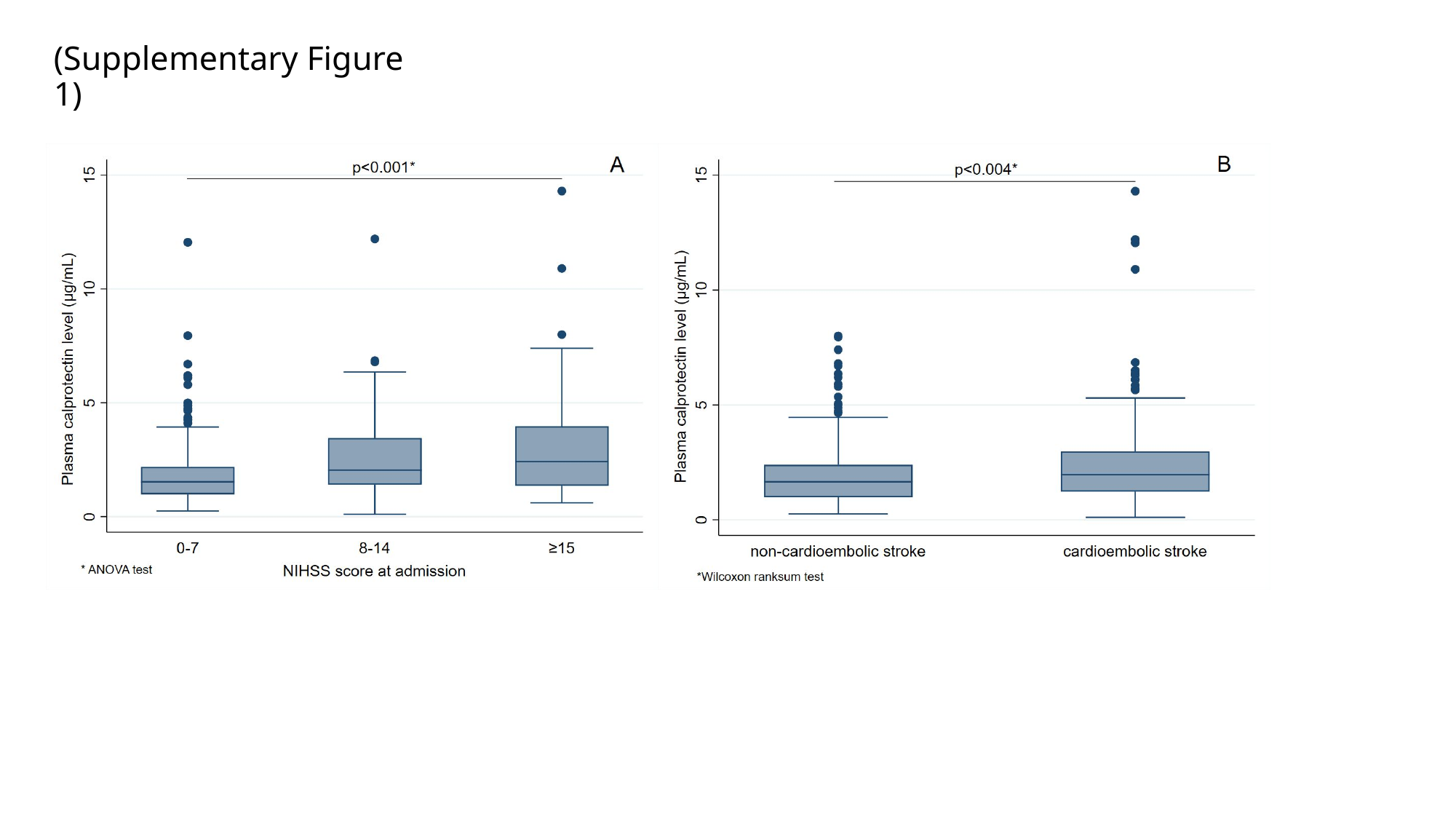

(Supplementary Figure 1)

Supplement: Supplementary file 1 — Additional file 1: Supplementary Figure 1. A, Association of NIHSS score with plasma calprotectin levels expressed by median calprotectin levels stratified by NIHSS score at admission in three categories. B, Differences in calprotectin levels between cardioembolic and non-cardioembolic strokes. NIHSS: National Institute of Health Stroke Scale. [file 12974_2020_2047_MOESM1_ESM.pptx]
