# Supplementary material for: Association of calprotectin with other inflammatory parameters in the prediction of mortality for ischemic stroke
Source: J Neuroinflammation. 2021 Jan 5;18:3. doi: 10.1186/s12974-020-02047-1 (PMC7786493; doi:10.1186/s12974-020-02047-1)
Supplement: Supplementary file 2 — Additional file 2: Supplementary Table 1. Univariate and forward-stepwise multivariate logistic regression model of 3-month mortality. Supplementary Table 2. Univariate and forward-stepwise multivariate logistic regression model of 3-month functional independence. Supplementary Table 3. Univariate and multivariate logistic regression models of hemorrhagic transformation after AIS with calprotectin and other baseline characteristics. Supplementary Table 4. Characteristics of stroke patients with thrombi retrieved by mechanical thrombectomy. [file 12974_2020_2047_MOESM2_ESM.docx]

**Supplementary Tables-**

**Supplementary Table 1**. Univariate and forward-stepwise multivariate logistic regression model of 3-month mortality.

|  | Univariate Logistic Regression | | Multivariate Logistic Regression (n=393) | |
| --- | --- | --- | --- | --- |
| Variable | OR (95%CI) | p value | OR (95%CI) | p value |
| Age (per 10-year increase) | 2.21(1.65-2.97) | <0.001 | 1.73 (1.18-2.53) | 0.005 |
| Sex (female) | 2.09 (1.23-3.56) | 0.007 | ƚ |  |
| Weight (Kg) * | 0.25 (0.06-1.03) | 0.055 | ƚ |  |
| Hypertension | 2.95 (1.41-6.18) | 0.004 | ƚ |  |
| Type-2 Diabetes | 0.64 (0.34-1.21) | 0.168 | ƚ |  |
| Dyslipidaemia | 0.66 (0.39-1.13) | 0.131 | ƚ |  |
| eGFR <45 ml/min/1.73m^2^ | 2.56 (1.22-5.37) | 0.013 | ƚ |  |
| SBP at admission *  (per 10mmHg increase) | 2.35 (0.48-11.48) | 0.290 |  |  |
| DBP at admission  (per 10mmHg increase) | 1.17 (1.00-1.36) | 0.048 | ƚ |  |
| Serum Glucose (mg/dL) * | 1.97 (0.88-4.44) | 0.101 | ƚ |  |
| Neutrophil count (_x_mm3) * | 3.64 (1.83-7.24) | <0.001 | ‡ |  |
| Lymphocyte count (_x_mm3) * | 0.42 (0.24-0.72) | 0.002 | ‡ |  |
| NLR * | 2.41 (1.63-3.58) | <0.001 | ƚ |  |
| C-Reactive Protein *  (mg/L) | 1.02 (1.01-1.03) | <0.001 | ƚ |  |
| Calprotectin µg/mL * | 4.60 (2.86-7.40) | <0.001 | 3.00 (1.71-5.26) | <0.001 |
| NIHSS score  8-14 (vs 0-7)  >14 (vs 0-7) | 3.75 (1.60-8.80) | 0.002 | 2.23 (0.83-6.02) | 0.112 |
|  | 11.86 (5.85-24.05) | <0.001 | 7.04 (3.08-16.10) | <0.001 |
| ASPECTS ≥7 | 0.08 (0.03-0.24) | <0.001 | 0.19 (0.05-0.77) | 0.020 |
| baseline mRS score | 2.10 (1.66-2.65) | <0.001 | 1.57 (1.14-2.15) | 0.005 |
| Intravenous thrombolysis | 2.33 (1.37-3.98) | 0.002 | ƚ |  |
| Endovascular treatment | 1.49 (0.77-2.89) | 0.240 |  |  |
| Hemorrhagic transformation | 4.04 (2.10-7.77) | <0.001 | ƚ |  |
| Cardioembolic stroke | 2.37 (1.38-4.06) | 0.002 | ƚ |  |

eGFR: estimated glomerular filtration rate; SBP: Systolic Blood Pressure; DBP: Diastolic Blood Pressure; NLR: Neutrophil-to-lymphocyte Ratio; NIHSS: National Institute of Health Stroke Scale; ASPECTS: Alberta Stroke Program Early CT Score; mRS: modified Rankin Scale.

* log transformed.

ƚ Excluded by forward stepwise selection (inclusion p<0.05 and exclusion p>0.10)

‡ Excluded to avoid collinearity between Neutrophils, Lymphocytes and NLR.

**Supplementary Table 2**. Univariate and forward-stepwise multivariate logistic regression model of 3-month functional independence.

|  | Univariate Logistic Regression | | Multivariate Logistic Regression (n=325) | |
| --- | --- | --- | --- | --- |
| Variable | OR (95%CI) | p value | OR (95%CI) | p value |
| Age (per 10-year increase) | 0.57 (0.46-0.71) | <0.001 | 0.58 (0.44-0.77) | <0.001 |
| Sex (female) | 0.67 (0.43-1.06) | 0.089 | ƚ |  |
| Weight (Kg) * | 0.87 (0.26-2.93) | 0.827 |  |  |
| Hypertension | 0.74 (0.45-1.21) | 0.232 |  |  |
| Type-2 Diabetes | 0.87 (0.53-1.42) | 0.572 |  |  |
| Dyslipidemia | 0.96 (0.61-1.50) | 0.852 |  |  |
| eGFR <45 ml/min/1.73m^2^ | 0.44 (0.20-0.97) | 0.041 | ƚ |  |
| SBP at admission *  (per 10mmHg increase) | 1.40 (0.36-5.36) | 0.628 |  |  |
| DBP at admission  (per 10mmHg increase) | 1.01 (0.89-1.16) | 0.830 |  |  |
| Serum Glucose at admission (mg/dL) * | 0.34 (0.16-0.71) | 0.004 | 0.24 (0.10-0.62) | 0.003 |
| Neutrophil count (_x_mm3) * | 0.37 (0.20-0.66) | 0.001 | ‡ |  |
| Lymphocyte count (_x_mm3) * | 2.40 (1.50-3.86) | <0.001 | ‡ |  |
| NLR * | 0.44 (0.31-0.62) | <0.001 | 0.50 (0.33-0.77) | 0.002 |
| C-Reactive Protein (mg/L) * | 0.64 (0.53-0.78) | <0.001 | ƚ |  |
| Calprotectin µg/mL * | 0.49 (0.34-0.70) | <0.001 | ƚ |  |
| NIHSS score at admission  8-14 (vs 0-7)  >14 (vs 0-7) | 0.16 (0.08-0.30) | <0.001 | 0.14 (0.07-0.30) | <0.001 |
|  | 0.12 (0.07-0.21) | <0.001 | 0.16 (0.07-0.32) | <0.001 |
| ASPECTS at baseline ≥7 | 18.95 (2.37-151.57) | 0.006 | ƚ |  |
| Baseline mRS score ≤1 | 2.39 (1.40-4.06) | 0.001 |  |  |
| Intravenous thrombolysis | 0.47 (0.30-0.74) | 0.001 | ƚ |  |
| Endovascular treatment | 0.51 (0.29-0.89) | 0.018 | ƚ |  |
| Hemorrhagic transformation | 0.13 (0.06-0.28) | <0.001 | 0.32 (0.13-0.83) | 0.018 |
| Cardioembolic stroke | 0.48 (0.32-0.73) | <0.001 | ƚ |  |

eGFR: estimated glomerular filtration rate; SBP: Systolic Blood Pressure; DBP: Diastolic Blood Pressure; NLR: Neutrophil-to-lymphocyte Ratio; NIHSS: National Institute of Health Stroke Scale; ASPECTS: Alberta Stroke Program Early CT Score; mRS: modified Rankin Scale.

* log transformed.

ƚ Excluded by forward stepwise selection (inclusion p<0.05 and exclusion p>0.10)

‡ Excluded by authors to avoid collinearity between Neutrophils, Lymphocytes and NLR.

**Supplementary Table 3.** Univariate and multivariate logistic regression models of hemorrhagic transformation after AIS with calprotectin and other baseline characteristics.

|  | Univariate Logistic Regression | | Multivariate Logistic Regression (n=385) | |
| --- | --- | --- | --- | --- |
| Variable | OR (95%CI) | p value | OR (95%CI) | p value |
| Age (per 10-year increase) | 1.04 (0.82-1.32) | 0.735 |  |  |
| Sex (female) | 0.52 (0.28-0.98) | 0.044 | 0.32 (0.13-0.75) | 0.009 |
| Weight (kg) * | 1.27 (0.24-6.61) | 0.775 |  |  |
| Hypertension | 0.77 (0.41-1.45) | 0.422 |  |  |
| Type-2 Diabetes | 0.62 (0.30-1.29) | 0.201 |  |  |
| Dyslipidaemia | 0.78 (0.43-1.40) | 0.399 |  |  |
| eGFR <45 ml/min/1.73m^2^ | 0.56 (0.17-1.90) | 0.356 |  |  |
| SBP at admission *  (per 10mmHg increase) | 0.44 (0.08-2.54) | 0.357 |  |  |
| DBP at admission  (per 10mmHg increase) | 0.98 (0.83-1.17) | 0.861 |  |  |
| Serum Glucose at admission (mg/dL) * | 1.18 (0.46-3.07) | 0.727 |  |  |
| Neutrophil count (_x_mm3) * | 1.91 (0.90-4.04) | 0.090 | ƚ |  |
| Lymphocyte count (_x_mm3) * | 0.70 (0.39-1.28) | 0.247 |  |  |
| NLR * | 1.49 (0.98-2.29) | 0.065 | 1.71 (0.94-3.10) | 0.076 |
| C-Reactive Protein (mg/L) * | 1.57 (1.23-2.00) | <0.001 | 1.19 (0.81-1.73) | 0.375 |
| Calprotectin µg/mL * | 1.67 (1.08-2.58) | 0.022 | 1.21 (0.60-2.41) | 0.598 |
| NIHSS score at admission  7-14 (vs 0-7)  >14 (vs 0-7) | 4.45 (1.44-13.74) | 0.009 | 1.09 (0.27-4.45) | 0.907 |
|  | 21.39 (8.66-52.89) | <0.001 | 6.02 (1.78-20.36) | 0.004 |
| ASPECTS at baseline ≥7 | 1.00 (0.22-4.55) | 0.997 |  |  |
| mRS score at baseline | 1.09 (0.84-1.41) | 0.504 |  |  |
| Intravenous thrombolysis | 14.98 (6.54-34.32) | <0.001 | 15.70 (4.40-55.97) | <0.001 |
| Endovascular treatment | 7.52 (3.99-14.16) | <0.001 | 2.26 (0.92-5.55) | 0.075 |
| Antiplatelet therapy | 0.91 (0.48-1.72) | 0.764 | 0.94 (0.38-2.27) | 0.883 |
| Anticoagulant therapy | 0.92 (0.44-1.92) | 0.819 | 1.92 (0.52-7.06) | 0.326 |
| Cardioembolic stroke | 2.07 (1,14-3.75) | 0.017 | 1.30 (0.53-3.17) | 0.563 |

eGFR: estimated glomerular filtration rate; SBP: Systolic Blood Pressure; DBP: Diastolic Blood Pressure; NLR: Neutrophil-to-lymphocyte Ratio; NIHSS: National Institute of Health Stroke Scale; ASPECTS: Alberta Stroke Program Early CT Score; mRS: modified Rankin Scale.

* log transformed.

ƚ Excluded by authors to avoid collinearity between Neutrophils and NLR.

**Supplementary Table 4.** Characteristics of stroke patients with thrombi retrieved by mechanical thrombectomy.

| Variable | n=44 |
| --- | --- |
| Age; years ƚ | 74.8 (61.7-78.0) |
| Female ‡ | 18 (40.9) |
| Hypertension ‡ | 26 (59.1) |
| Type-2 Diabetes ‡ | 7 (15.9) |
| Dyslipidemia ‡ | 25 (56.8) |
| Antiplatelet therapy ‡ | 10 (22.7) |
| Anticoagulant therapy ‡ | 13 (29.6) |
| SBP at admission, mmHg * | 142 (21.7) |
| DBP at admission, mmHg * | 82.7 (14.9) |
| Serum glucose at admission, mg/dl ƚ | 110 (96-125) |
| Neutrophil count at admission, x10^9^/L ƚ | 5.8 (4.4-7.8) |
| Lymphocyte count at admission, x10^9^/L ƚ | 1.7 (1.3-2.5) |
| Baseline NIHSS score ƚ | 18 (16-21) |
| ASPECTS <7 ‡ | 0 (0) |
| Baseline mRS score ‡ |  |
| mRS 0 | 29 (65.9) |
| mRS 1 | 8 (18.2) |
| mRS 2 | 7 (15.9) |
| Intravenous thrombolysis ‡ | 34 (77.3) |
| Hemorrhagic transformation ‡ | 17 (38.6) |
| 3-month mortality ‡ | 12 (27.9) |
| 3-month functional independence ‡ | 17 (39.5) |
| Cardioembolic stroke ‡ | 28 (63.6) |
| Atherothrombotic stroke ‡ | 6 (13.6) |

SD: standard deviation; IQR: interquartile range (denoted by 25^th^-75^th^ percentile); SBP: Systolic Blood Pressure; DBP: Diastolic Blood Pressure; NIHSS: National Institute of Health Stroke Scale; ASPECTS: Alberta Stroke Program Early CT Score; mRS: modified Rankin Scale.

* Continuous variables with normal distributions are presented as means (SD).

ƚ Continuous non-normally distributed variables are presented as medians (IQR).

‡ Categorical variables are presented as n (%).
